# Carbon Quantum Dots from Amino Acids Revisited: Survey of Renewable Precursors toward High Quantum-Yield Blue and Green Fluorescence

**DOI:** 10.1021/acsomega.2c04751

**Published:** 2022-11-01

**Authors:** Anna Kolanowska, Grzegorz Dzido, Maciej Krzywiecki, Mateusz M. Tomczyk, Dariusz Łukowiec, Szymon Ruczka, Sławomir Boncel

**Affiliations:** † Faculty of Chemistry, Department of Organic Chemistry, Bioorganic Chemistry and Biotechnology, Silesian University of Technology, Krzywosutego 4, 44-100Gliwice, Poland; ‡ Faculty of Chemistry, Department of Physical Chemistry and Technology of Polymers, Silesian University of Technology, Strzody 9, 44-100Gliwice, Poland; § Biotechnology Centre, Silesian University of Technology, Krzywoustego 8, 44-100Gliwice, Poland; ∥ Faculty of Chemistry, Department of Chemical Engineering and Process Design, Silesian University of Technology, Strzody 7, 44-100Gliwice, Poland; ⊥ Institute of Physics—CSE, Silesian University of Technology, Konarskiego 22B, 44-100Gliwice, Poland; # Materials Research Laboratory, Faculty of Mechanical Engineering, Silesian University of Technology, Konarskiego 18A, 44-100Gliwice, Poland; ¶ Centre for Organic and Nanohybrid Electronics, Silesian University of Technology, Konarskiego 22B, 44-100Gliwice, Poland

## Abstract

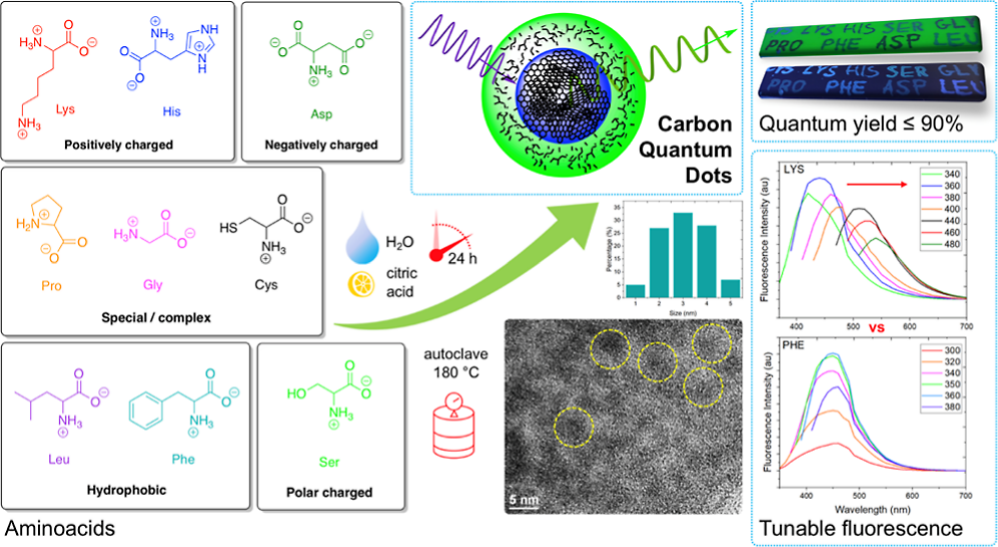

Carbon quantum dots (CQDs) were synthesized via a green, one-step hydrothermal method. As CQD precursors, nine amino acids of different structural descriptors (negatively/positively charged in water, polar, hydrophobic, sulfur-containing, and other/complex ones) were surveyed: Asp, Cys, Gly, His, Leu, Lys, Phe, Pro, and Ser. The reactions were performed in an autoclave in the presence of citric acid at 180 °C for 24 h and yielded core–shell CQDs. CQDs were comprehensively characterized by transmission electron microscopy, dynamic light scattering, Raman, UV/Vis, infrared, X-ray photoelectron spectroscopy, and fluorescence spectroscopy. At the excitation wavelength of λ_ex_ = 350 nm, Cys-, Phe-, Leu-, and Lys-based CQDs displayed the highest quantum yield blue fluorescence—90 ± 5, 90 ± 4, 87 ± 5, and 67 ± 3%, respectively—superior to the conventional fluorescent dyes. Strikingly, for Lys- and Phe-CQDs, dissimilar trends in the excitation–emission wavelength relationships were identified, that is, constantly strong red shifts versus excitation wavelength-independent emission. Cys- and Lys-CQDs were water-dispersible toward the narrow unimodal distribution of hydrodynamic diameters—0.6 and 2.5 nm, respectively. Additionally, Lys- and Cys-CQDs, with high absolute zeta potential values, formed stable aqueous colloids in a broad range of pH (2, 7, and 12). The results constitute important premises for water-based applications of CQDs, such as bioimaging or photocatalysis.

## Introduction

Carbon quantum dots (CQDs) are fluorescent nanomolecules or nanoparticles smaller than 10 nm.^1,2^ The most studied 0D CQDs consist of a more graphitized, sp^2^-carbon rich core surrounded with a 5–50 wt % amorphous shell of polar functional groups.^3−6^ The structure of CQDs results in their excellent solubility in water, negligible cytotoxicity, and biocompatibility,^7^ and while bearing carboxylic groups, CQDs are conveniently functionalizable.^8^ CQDs combine the unique optical properties of quantum dots (QDs) with the electronic properties of carbon (nano)materials. Importantly, CQDs display a quantum limitation effect, translating into tunable absorption and emission.^9^ It means that after excitation, the energy of the emitted photons depends on the CQD size and its molecular structure. Hence, small CQDs fluoresce in blue while the emission wavelengths increase, with the CQD diameter spanning the entire range of visible light up to infrared (IR).^10^ Theoretical calculations of the emission wavelength of pristine zigzag-edged CQDs showed that CQDs of diameters 0.5 and 2.31 nm fluoresced at 235.2 to 999.5 nm wavelengths, respectively.^11^ At the same time, altering the core–shell CQD composition by N-, S-, P-, or B-doping enhances the fluorescence quantum yield (QY). Such doping alters the energy between the lowest unoccupied (LUMO) and the highest occupied molecular orbitals (HOMO).^12^ By the interplay of CQD chemistry and morphology, it is possible to obtain fluorescence in the full spectral range from ultraviolet (UV) to near-IR (NIR). With the above characteristics, CQDs emerge as promising biosensors,^13^ imaging agents,^14^ and drug delivery systems,^15^ along with multi-modality. Due to their high electron mobility, long hot-electron lifetimes, ultrafast electron extraction, tunable band gaps, excellent electron donor/acceptor properties, and strong stable fluorescence, CQDs are considered as photocatalysts^16,17^ and working elements in optoelectronic devices.^18^

CQDs can be synthesized via a bottom-up approach from renewable sources such as fruit and vegetable peels,^19−22^ nuts,^23−25^ wastes,^26,27^ or larger carbon (nano)materials in the top-down methods.^28,29^ CQDs containing mainly carbon and oxygen (and hydrogen) suffer from low QY, while N-doping is the most frequently applied strategy to improve fluorescence. This modification introduces structural defects and new energy states and increases the number of electrons in the conduction band. Therefore, well-defined, small-molecule amino acids (AAs) emerge as promising candidates for the synthetic precursors of CQDs. AAs are renewable, abundant (global volume of the AA market reached 10.3 MT in 2021), relatively inexpensive (110–1300 USD kg^–1^), and non-toxic.^30^ Zwitter-ionic and polyfunctional AAs can be variously charged depending on pH and equipped with hydrophilic (hydroxy −OH or mercapto −SH groups) or hydrophobic (aliphatic and/or aromatic) moieties, which, in turn, provides tunability of the optical properties of CQDs.^31,32^

Here, we propose a facile and sustainable one-step hydrothermal synthesis of CQDs from various AAs (hydrophilic, hydrophobic, aromatic, and aliphatic) and citric acid (CA) as the precursors of the core and shell, respectively. Our method covers a fully controlled four-stage synthesis, that is, dehydration, polymerization, passivation, and carbonization. The as-synthesized CQDs exhibit blue to green fluorescence—exhibiting red-shifts depending on the synthetic precursor—with merits of narrow size distribution and excellent water solubility, while the excitation wavelength falls in the range of 300 to 480 nm. Importantly, using cystein, phenylalanine, and leucine—as the synthetic precursors under the optimized conditions—we show that it is possible to obtain CQDs with high QYs superior to the conventional fluorescent dyes. Lys-CQDs emerged as also forming stable aqueous dispersions in a broad range of pH. The overall characteristics allow us to address the key prerequisites for numerous applications.

## Materials and Methods

### Materials

#### Synthesis of CQDs

CA, quinine sulfate (QS), 7-diethylamino-4-methylcoumarin (Coumarin 1), and AAs were purchased from Sigma-Aldrich. CQDs were synthesized using a one-step hydrothermal method. CA (1.5 mmol) and AA (aspartic acid, cysteine, glycine, histidine, leucine, lysine, phenylalanine, proline, and serine) (1.5 mmol) were dissolved in distilled water (10 mL). The amount of water was adjusted to dissolve CA and AA at room temperature. The solution was heated in a Teflon-coated autoclave at 180 °C for 24 h in a laboratory dryer. The autoclave was allowed to cool down to room temperature, and the post-reaction mixture was centrifuged at 5500 rpm for 15 min to separate the larger particles. The resulting supernatant was filtered through a 0.22 μm-syringe filter (Minisart NY hydrophilic polyamide, 25 mm). Following purification, the solution was frozen in liquid nitrogen and lyophilized until dried.

### Instrumentation

The characterization of CQDs was performed by transmission electron microscopy (TEM) (S/TEM Titan 80–300 operated at 300 kV, Field Electron and Ion Company), combustional elemental analysis (PerkinElmer 2400 Series II CHNS/O, PerkinElmer), thermogravimetric analysis (TGA) (TGA 8000, PerkinElmer), Raman (inVia Confocal Raman microscope, Renishaw), UV–Vis (HP 8452A UV–Vis Diode Array Spectrophotometer, Hewlett Packard), fluorescence spectroscopy (SpectraMax i3x, Molecular Devices and FluoroMax Plus, Horiba Scientific), Fourier-transform IR (FT-IR) (Nicolet 6700 FT-IR, Thermo Fischer Scientific), and X-ray photoelectron spectroscopy (XPS) (PreVac EA15, PreVac). Additionally, by applying dynamic light scattering (DLS), nanoparticle size and zeta-potential were determined (Zetasizer Nano S90, Malvern Panalytical).

### Transmission Electron Microscopy

The nanomorphology of CQDs was determined based on TEM images collected using a transmission electron microscope S/TEM TITAN 80–300. The samples were prepared by dispersion and ultrasonication of CQDs in ultrapure ethanol and then placed on a copper TEM grid with lacey carbon films (200 mesh).

### Combustional Elemental Analysis

A sample of CQDs (ca. 2–10 mg) was accurately weighed into small tin capsules. At elevated temperatures, in the presence of excess oxygen, the organic materials combusted into CO_2_, H_2_O, SO_2_, and N_*x*_O_*y*_ compounds (next reduced by fine copper particles in the reduction tube to N_2_). For quantitative analysis, CO_2_, H_2_O, SO_2_, and N_2_ content represent carbon, hydrogen, sulfur, and nitrogen content, respectively. Oxygen content was calculated indirectly from the difference between the sample weight and the sum of the other element contents.

### TGA Analysis

TGA curves were acquired under nitrogen (flow rate of 40 mL min^–1^). The samples (1–5 mg) were heated in alumina crucibles up to 800 °C at a heating rate of 20 °C min^–1^.

### Raman Spectroscopy

Raman spectra were obtained at 514 nm (a green laser) with a laser power of 5%, a 2400 line per mm grating, 20× magnification, and an exposure time of 15 s. For each material, three accumulations were collected in three locations within the sample. The spectra were averaged and normalized to the G-band.

### FT-IR Spectroscopy

Spectra were collected in the range of 400–4000 cm^–1^, with 16 scans for each sample with a resolution of 4 cm^–1^. Lyophilized CQDs were mixed with dry KBr in an agate mortar and then pressed in an evacuable slot to form a pellet under 40 MPa pressure for 2 min using a hydraulic press.

### X-ray Photoelectron Spectroscopy

XPS measurements were performed in a UHV multi-chamber experimental setup with a PreVac EA15 hemispherical electron energy analyzer fitted with a 2D multi-channel plate detector. The system base pressure was equal to 9 × 10^–9^ Pa. An Mg-Kα X-ray source (PreVac dual-anode XR-40B source, excitation energy of 1253.60 eV) was used to excite the sample. Pass energy was set to 200 eV for the survey spectra collection (scanning step of 0.9 eV) and to 100 eV for high-accuracy energy regions (scanning step of 0.06 eV). All measurements were done with a normal take-off angle and the curved analyzer exit slit (0.8 × 25 mm) choice for the highest energy resolution. The binding energy scale of the analyzer was calibrated to the Au 4f_7/2_ (84.0 eV) region of the gold-covered sample placed at the same sample stage.^33^ The acquired spectra were fitted using CasaXPS software. The components were fitted with the sum of Gauss (30%) and Lorenz (70%) functions, while the Shirley function was applied for background subtraction.

### UV–Vis Spectroscopy

UV–Vis spectra were obtained in quartz cuvettes (2 mL) with a 10 mm optical path at a scanning rate of 1.0 nm from 250 to 800 nm.

### Fluorescence Spectroscopy

The fluorescence spectra were measured under different excitation wavelengths (from 250 to 480 nm) for 200 μL of the sample transferred to a clear bottom 96-well plate (scan speed 20 nm min^–1^).

The QY (φ) of CQDs was calculated using QS (φ = 54%) in 0.1 M H_2_SO_4(aq)_ and Coumarin 1 (φ = 59%) in ethanol as the references by comparing the integrated photoluminescence intensity and absorbance.^34,35^ Samples of aqueous CQD suspensions of different concentrations were prepared by keeping the absorbance values less than 0.1 at their excitation wavelengths (similar to different CQD concentrations). Next, the integrated photoluminescence intensities for all samples were measured. The integrated photoluminescence intensity was plotted against absorbance, and the slope values of the obtained linear plots were measured. The QY was calculated using the below equation
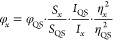
where: φ—QY; *S*—integrated fluorescence intensity (area under spectrum); *I*—fluorescence intensity; η—refractive index; and *x*—CQD sample.

### DLS Measurement

The hydrodynamic diameter and zeta potential of CQDs were measured by DLS using a monochromatic coherent He–Ne laser with a fixed wavelength of 633 nm. The measurements were performed in triplicate for 2 mL of sample (1 mg mL^–1^) in distilled water. The zeta potential for each sample was measured for three pH values: 2.0, 7.0, and 12.0. The pH of the suspension was adjusted by adding HCl_(aq)_ or NaOH_(aq)_.

## Results and Discussion

**Figure 1:**
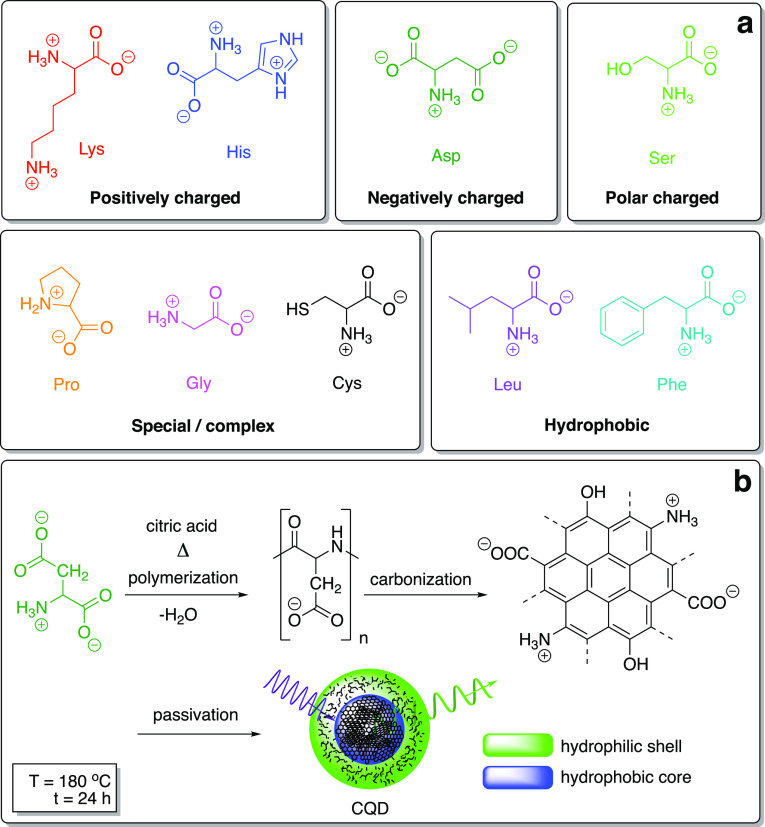
Skeletal molecular formulae of AAs with different structural descriptors as the CQD precursors, including the net charge in water (a) and the general synthetic pathway toward CQDs—here illustrated by the hydrothermal transformation of Asp via the four-stage decomposition (b).

The molecular structure of the AA substrates and the conditions represent the most important variables in the properties-by-design synthesis of CQDs. As optimized, white to yellowish mat CQD powders were synthesized via the hydrothermal method, lasting 24 h at 180 °C—employing as substrates nine different AAs and CAs (as the main carbon core precursor) (Figure 1). Our synthetic protocol was inspired by numerous earlier studies. For instance, Chahal et al. proved that the application of both CA and AAs is necessary for higher yields in the CQD synthesis, displaying high QYs.^36^ Indeed, in the absence of CA, the synthesis of CQDs proceeds at low yields. The authors claimed that CA played two roles in the CQD preparation. First, CA emerged as a multifunctional compound bearing three carboxyl groups and one hydroxyl group, indicating several sites to react with AAs and also with other CA molecules. Second, CA served as a Brønsted acidic catalyst in the addition–elimination reactions.

Here, the rationale behind the selection of AAs was to cover their most important structural descriptors (Figure 1a). The CQD products of the synthesis from the particular AA (in the form of three-letter international codes) are denoted as AA-CQDs such as, for example, Phe-CQD, representing l-phenylalanine-derived CQDs. The unique colors of molecular formulae of AAs are consequently applied in the analyses and spectra throughout the entire work for the sake of clarity and unambiguity.

AAs bear amino and carboxylic acid groups, enabling the formation of a variety of nitrogen and oxygen functionalities within the CQD shell, while Cys also provides sulfur moieties. Upon hydrothermal synthesis, the AA molecules first assemble as a result of hydrogen bonding. Next, upon heating and subsequent dehydration, polymerization occurs, leading to a short single burst of nucleation. The resulting nuclei grow by the diffusion of solutes toward CQD surfaces.^37^ Such a mechanism describing the synthetic route for the “bottom-up” methods has been proposed by many researchers. Accordingly, synthesis of CQDs includes polymerization (polycondensation via dehydration), nucleation, carbonization, and growth. In our attempt, the polymer carbon skeleton is proposed as a cross-linking agent after dehydration, whereas upon carbonization, a fraction of the precursors is consumed to further modify the carbon core.^38^

**Figure 2:**
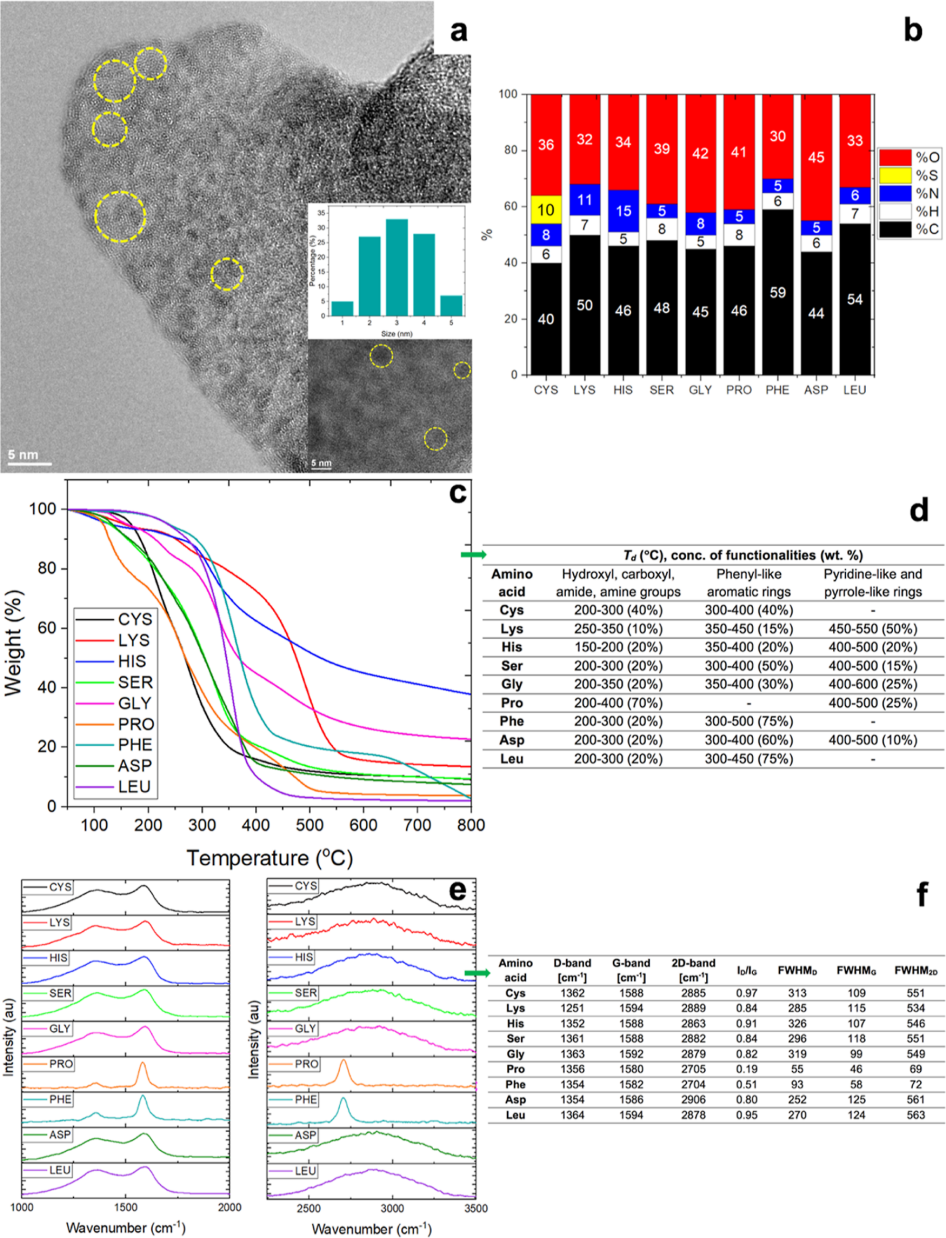
Structural features of the as-synthesized CQDs. TEM images of Phe-CQDs; top inset shows the particle size distribution estimated from 100 individual CQDs, and the bottom inset presents a higher magnification image showing isolated Phe-CQDs (a); combustion elemental analysis of CQDs (b); TGA curves of CQDs (c); analysis of the corresponding step-wise thermal degradation of CQDs (d); Raman spectra of CQDs at the critical regions: D-, G-, and 2D-bands (e); summary of the key Raman spectra parameters for all CQDs (f). FWHM—full width at half maximum—for D-, G-, and 2D-peaks.

The general morphological, compositional, and structural features of the as-synthesized CQDs were analyzed (Figure 2). The morphology and size distribution of the as-synthesized CQDs were analyzed by TEM (Figure S1). The imaging showed that CQDs were composed predominantly of quasi-spherical and amorphous shells (revealing a “halo-effect” at the CQD edges that are less graphitized and hence rich in sp^3^-carbon atoms). The size distribution of Phe-CQDs, further selected as one of the most perspective ones in terms of high QY, was narrow, that is, in the range of 1–5 nm with the abundance peak at 3 nm (as determined from the population of 100 CQDs) (Figure 2a and the insets). TEM images of other CQDs frequently showed nanoparticle agglomerates larger than 10 nm, presumably formed upon lyofilization. The selected area electron diffraction (SAED) patterns of CQDs were primarily composed of diffused rings (Figure S2). This behavior stayed in good agreement with the literature data—CQDs prepared using hydrothermal methods were generally found to be amorphous.^39^ Nevertheless, SAED analysis also revealed diffraction spots assignable to the polycrystalline graphitic areas (Figure S2). Furthermore, we have performed combustion elemental analysis of CQDs (Figure 2b), which were found to be composed mainly of carbon (from 39 wt % for Cys-CQDs to 59 wt % for the more graphitized Phe-CQDs). For all CQDs, the shell surface was rich in oxygen and nitrogen functional groups. The elemental oxygen content varied from 30 wt % for Phe-CQDs to 44 wt % for Asp-CQDs. In turn, the highest nitrogen content was observed for His-CQDs (15 wt %) with the lowest one for Pro-CQDs (4.5 wt %), which corresponds to the less pronounced gasification of the aromatic moieties for His-CQDs upon the synthesis. As predicted, in the case of Cys-CQDs, apart from carbon, oxygen, and nitrogen atoms, CQDs contained sulfur, although at as high as 10 wt % content.

TGA was applied to indirectly trace the chemical nature of CQDs via thermal degradation under pyrolytic conditions (Figure 2c). Depending on the precursor, CQDs are decomposed in two or three steps (Figure 2d). The weight loss below 200 °C corresponds to the moisture evaporation, dehydration (including constitutional water), and the evolution of pyrogases (CO_2_, CO, etc.) from the CQD surface. The losses in the range of 200–350 °C match the evolution of gasification products from different functional groups (hydroxyl, carboxyl, carbonyl, amide, and amine groups) from the exteriors (cores) of CQDs.^40^ The decomposition of the carbonaceous material occurred in the range of 300–450 °C, while scission of the aromatic nitrogen functionalities began with a plateau-like run above 550 °C;^41^ indeed, no further degradation was observed onward. The brownish to black residue content corresponded to the highly carbonized, polyaromatic core with the highest weight percentages for His- (∼40 wt %), Lys-, Ser-, and Gly-derived CQDs (all ∼20 wt %).

To gain further insights into the chemistry of CQDs, Raman spectra were acquired and divided into two distinctly different regions (Figure 2e,f). The spectra showed typical graphitic features: (a) D-mode (disorder) (1350–1364 cm^–1^) activated by symmetry breaking at defects and edges, (b) G-band (graphitic) (1580–1594 cm^–1^) arising from the in-plane C–C deformations, and (c) the second order features corresponding to 2D, D + G, and 2G combination modes.^42^ In all cases, the intensity of the G-band was higher than that of the D-band, indicating the dominating abundance of sp^2^-, with an addition of sp^3^-carbon atoms. CQDs can thus be considered as composed of sp^2^-graphitic and sp^2^ C=O carbon atoms (COOH, COO, CONH, etc.), with the admixture of sp^3^-carbon defects, including sp^3^-carbon-based functionalities (>CHOH, >CHO–, >CHNH–, etc.). The *I*_D_/*I*_G_ ratio identifies the nature of carbon atoms in CQDs and is typically used to determine the average size of the sp^2^-graphitic domains in carbon (nano)materials. The highest I_D_/I_G_ ratio, and as a consequence, the highest functionalization degrees/structural disorders and high N- and S-doping levels were observed for Cys- (0.97), Leu- (0.95), and His-CQDs (0.91). In turn, the lowest *I*_D_/*I*_G_ values were observed for Pro- (0.19) and Phe-CQDs (0.51) as the most ordered, that is, the least defective, hence resulting in a more aromatic/conjugated structure. Raman spectra were dependent on the measurement nanoscale position on the sample, indicating that the samples were a mixture of CQDs with different sizes and functionalization degrees. Indeed, for all CQDs, both D- and G-band frequencies have shown size-dependent trends, each one redshifted with the CQD size. The lowest G-frequency was observed for Pro-CQDs; however, it was not connected with the lowest D-frequency. This behavior may again correspond to the inhomogeneous distribution of CQD sizes. The lowest D-frequencies were observed for Lys- and His-CQDs. The differences between D- and G-frequencies were found to be higher than for Pro-CQD, which could prove that the homogeneity of size distribution for the latter one is lower. The highest D- and G-frequencies were observed for Leu-CQDs, which suggests the presence of the smallest CQDs. 2D-Band, which is the second-order D-band, is broader and blueshifted with the CQD size. This feature is more sensitive to the carbon core size, while the shift in D- and G-frequencies is the effect of not only the carbon core size but is also connected with higher functionalizations at the CQD core.^43^ Therefore, we can speculate that the Leu-CQD sample—featuring high D-, G-, and 2D frequencies—is composed of the medium size core and low molecular-weight dangling functional groups. In turn, the Phe-CQD sample with low D-, G-, and 2D frequencies contains CQDs with a small core and functional groups of higher molecular weight.^43,44^

**Figure 3:**
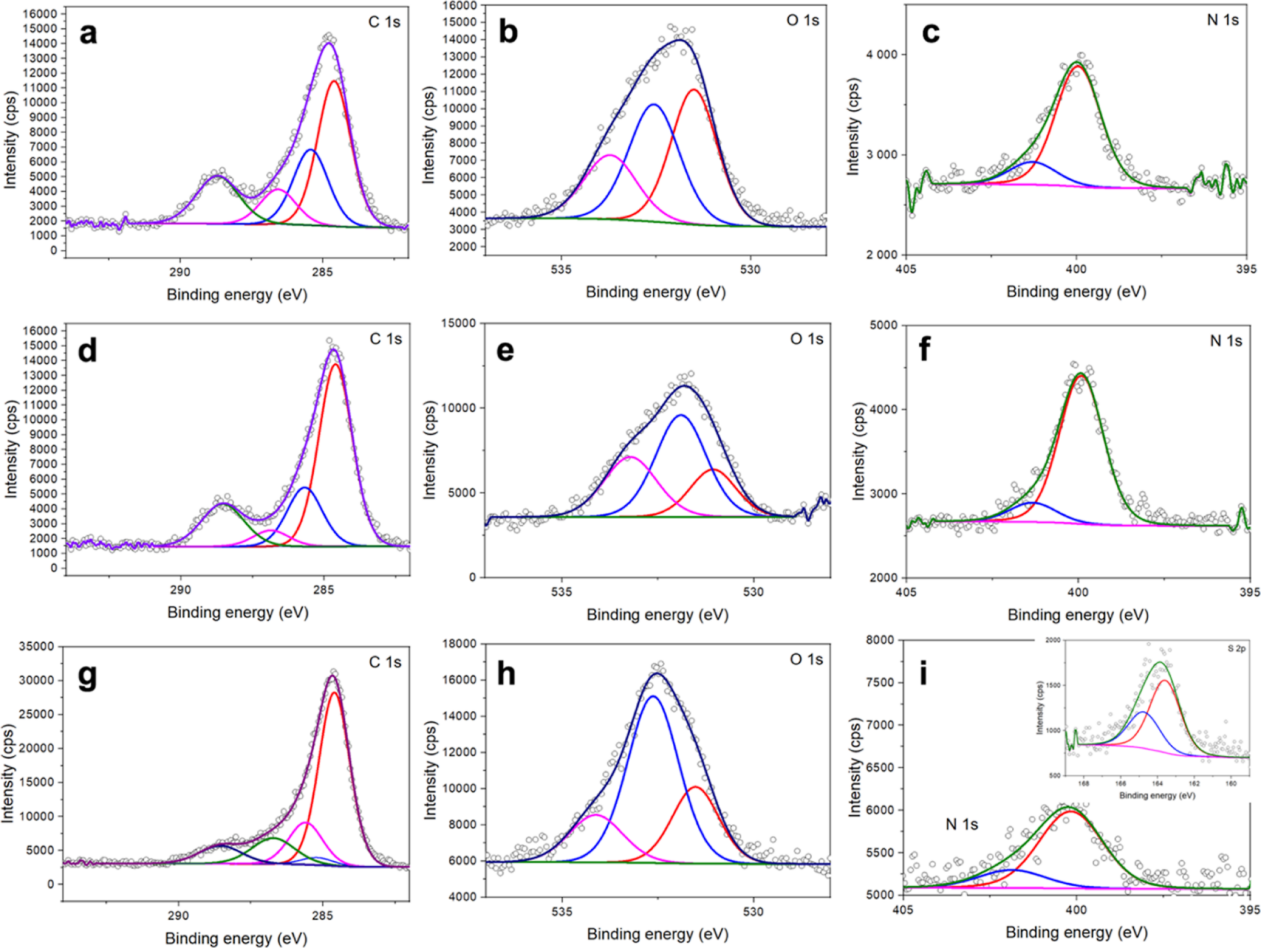
XPS spectra of CQDs. Signals and their deconvolution for Asp-CQDs in the C 1s (a), O 1s (b), and N 1s BE regions (c). Signals and their deconvolution for Leu-CQDs in the C 1s (d), O 1s (e), and N 1s BE regions (f). Signals and their deconvolution for Cys-CQDs in the C 1s (g), O 1s (h), N 1s (i), and S 2p BE regions (inset in i).

Figure 3 shows the decomposed XPS spectra of CQDs, most potentially from the applicability point-of-view. Figure 3a–c display XPS spectra of Asp-CQDs. Figure 3a shows the peak of photoemission for C 1s with the main peak for the carbon atoms located at a bonding energy (BE) of ca. 285 eV. Due to the presence of sp^2+ε^-carbon atoms, the peak is broad with a long asymmetric tail toward higher BE values.^45^ With the effect of functionalization, the concentration of sp^3^-carbon atoms increased, which resulted in the symmetric peak at 285.5 eV. The peaks corresponding to C–N/C–C=O/CONH_2_ (286.5 eV), C=O (287.5 eV), and COOH (288.5 eV) bonds/moieties could be assigned to the CQD surface functionalities.^45,46^Figure 3b shows XPS spectra obtained in the O 1s BE region with three key peaks at 531, 532.5, and 534 eV. The peak related to the COOH and OH is observed at a BE of 534 eV, while the one attributable to CO and CONH bonds appears at 532.5 nm. The strong peak at 531 eV can be assigned to C=O bonds.^45,47^Figure 3c shows the XPS in the N 1s BE region. The occurrence of the N 1s peak at 400 eV indicated the presence of CN/CONH_2_ groups,^45,47^ while the presence of C–N bonds was demonstrated in the region of C 1s peak at 286.5 eV. The weak peak at 401.5 eV can be assigned to N–H bonds present in the cationic moieties.^45^ Similarly, Figure 3d–f show XPS spectra for Leu-CQDs. The C 1 s spectrum (Figure 3d) consists of four contributions: 284.5, 285.5, 287.0, and 288.5 eV. The first and main contribution at 284.5 eV can be assigned to the graphitic carbon atoms. The contributions at 285.5, 287, and 288.5 eV are due to the presence of C–N/O=C–C/CONH_2_, C=O, and COOH moieties, respectively.^45,47^Figure 3e shows the peak of photoemission for O 1s with three key peaks at 531, 532, and 533.5 eV. The peak related to the COOH and OH is observed at a BE of 534 eV, while the one attributable to O–C and CONH_2_ bonds appears at 532 eV. The weak peak at 531.0 eV can be assigned to C=O bonds.^45^Figure 3f shows the XPS in the N 1s BE region, with the peaks at 400.0 and 401.5 eV belonging to N–C/CONH_2_ and N–H moieties, respectively.^45,47^ XPS spectra shown in Figure 3g–i clearly revealed that carbon, nitrogen, sulfur, and oxygen are present at the Cys-CQD surface. In the decomposed XPS spectra, the C 1s peaks at 284, 285, 285.5, 286.5, and 288.5 eV shown in Figure 3g can be assigned to carbon in the form of C–C/C–H, C–S, C–N/O=C–C/CONH_2_ C=O, and COOH.^45,47^ The O 1s peaks (Figure 3h) at 531.0, 532.5, and 534.0 eV are associated with oxygen in the states of O=C, O–C/CONH_2_, and COOH/OH, respectively.^45^ The N 1s peaks at 400 and 402 eV shown in Figure 3i indicate that nitrogen occurs mostly in the form of N–C/CONH_2_ and N–H. The S 2p spectrum in Figure 3i (inset) shows a broad peak at ∼164 eV, originating from C–S and S–H bonds with their spin–orbit splitting (separation of 1.18 eV) counterparts.^48,49^

**Figure 4:**
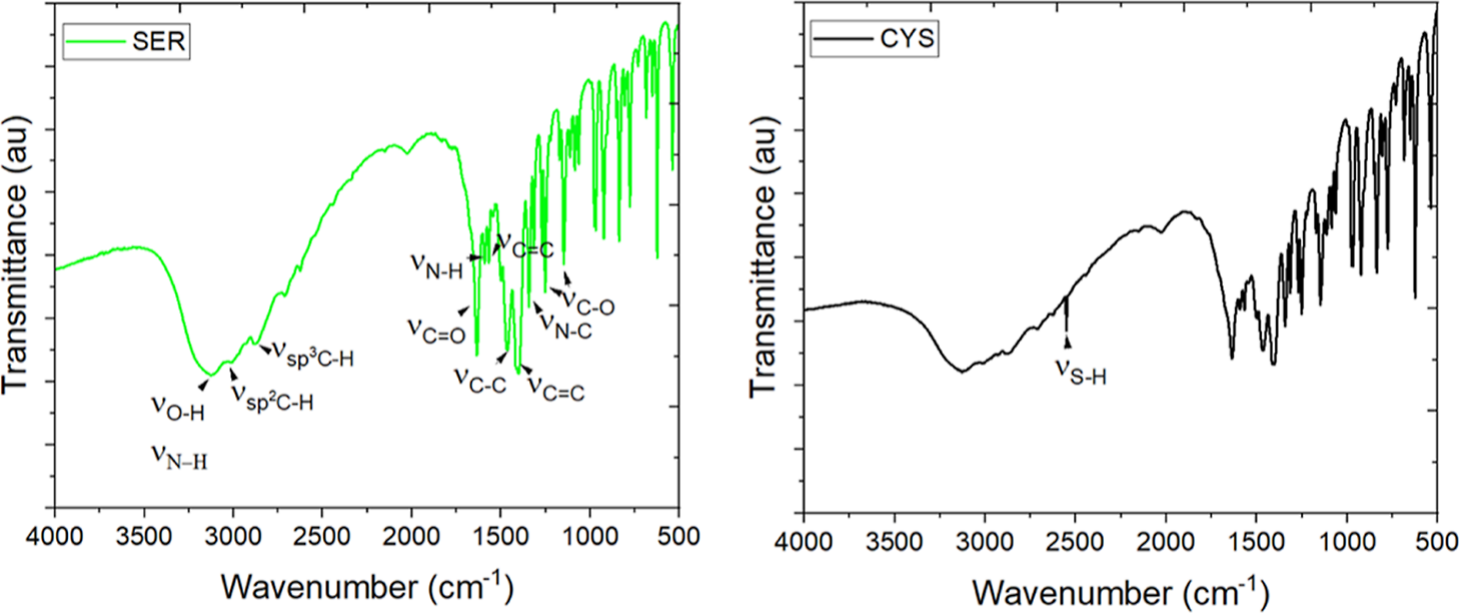
FT-IR spectra of Ser- (left) and Cys-CQDs (right).

FT-IR spectra (Figure 4) were used to further identify the functionalities in CQDs. The broad band in the range 3000–3500 cm^–1^ can be attributed to stretching vibrations of O–H^50^ and N–H^51^ groups. The band at 3042 cm^–1^ corresponds to the stretching vibrations of C–H in the aromatic species^52^ while at 2966 cm^–1^ in the aliphatic species.^53^ The strong absorption band at 1636 cm^–1^ corresponds to the stretching vibrations of carbonyl (C=O) groups.^54^ The absorption peak at 1591 cm^–1^ belongs to the C–N stretching vibration,^55^ while the peaks at 1570, 1467, and 1494 and 1340 cm^–1^ can be assigned to the stretching vibrations of C=C and bending vibrations of C–H.^54^ The bands appear at 1314, 1255, and 1143 cm^–1^, indicating the presence of the C–O stretching mode and the bending vibrations of NH_2_.^53,56,57^ Those bands (with only small shifts) are observed for all CQD samples (Figure S3). The presence of the C=C peak indicates that CQDs could also be composed of a fraction of the polycrystalline graphitic domains (referring back to SAED, Figure S2), whereas the other signals were assignable to −OH, C=O, C–N, N–H, and C–H functionalities. Many different vibrations were also found in the fingerprint regions, including C–O, C–N, C–C bond stretches, and C–H deformation vibrations. For Cys-CQDs, a specific but relatively weak band at 2551 cm^–1^ appears, conforming to the stretching vibrations of S–H bonds.^58^ Importantly, the concentration of the functional groups affects the fluorescence properties. Moieties like −CO and −COOH can reduce the energy gap and, therefore, red-shift the emission wavelength and reduce the QY. On the other hand, −OH groups can stabilize the surface sites, hence increasing the QY. Amino groups act as donors, transferring the electrons to the carbon core and stabilizing the emissive energy traps, increasing the QY.^59^

The optical properties of CQDs were evaluated by UV–Vis and fluorescence spectroscopy. All CQDs exhibited a strong absorption shoulder at 220–230 nm attributed to π–π* electron transition of the aromatic domains in the C=C and C=N bonds. In addition, the peaks at 300 nm are due to the n−π* transition in the π-conjugated structure. As all types of CQDs displayed a slight absorption at 350 nm due to the n−π* electron transition of the C=O groups, the fluorescence spectra were recorded for different excitation wavelengths. Upon UV irradiation (λ = 365 nm), bright fluorescence was observed for all CQDs, and the color of the CQD dispersions changed from yellowish to bright blue. Depending on the CQD precursor, the excitation–emission spectra typically showed a strong red-shift due to differences in the degree of surface oxidation and also an increase in the number of surface defects (Figure S4).^60^ Along with a change in the AA precursor, the photoluminescence peak of CQDs shifted from approximately 420 nm for Leu-CQDs to 450 nm for Phe-CQDs (for the excitation wavelength of 350 nm); yet, FWHM is rather high.

**Figure 5:**
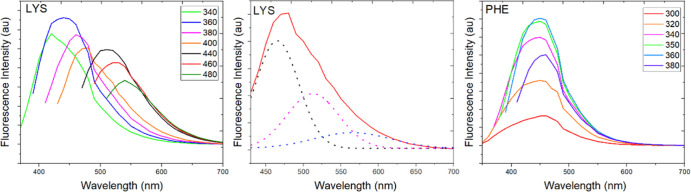
Fluorescence emission spectra of Lys- (left) with its exemplary (λ_ex_ = 400 nm) deconvolution (middle) and Phe-CQDs (right) at different excitation wavelengths (ranging from 300 to 480 nm).

For most samples, for example, Lys-CQDs (Figure 5, left), the emission wavelength could be related to the excitation wavelength; that is, along with the changed excitation wavelength from 300 to 480 nm. The photoluminescence peak of the Lys-CQDs was constantly red-shifted.

This excitation-dependent photoluminescence behavior has been extensively reported in fluorescent carbon-based nanomaterials, which might be due to the optical selection of differently sized CQDs and the interactions between the surface functionalities and the C-sp^2^-core.^61^ A completely different scenario was observed for Phe-CQDs (Figure 5, right), which showed changes in the fluorescence intensity when the excitation wavelength was increased, while the position of the fluorescence peak was redshifted, but to a constant position. Moreover, the spectra for excitation-dependent Lys-CQDs exhibited a broader emission peak than for the excitation-wavelength-independent Phe-CQDs. Deconvolution of the fluorescence spectra of Lys-CQDs (Figure 5, middle) revealed that the broad bands could actually be the combination of two or more bands with different fluorescence maxima. This phenomenon again confirms that one deals with a mixture of CQDs, and there are more types of excitation energies trapped on the surface of CQDs.

QY has been measured following the reported protocols using QS as the reference (Table 1). QY can be correlated with the chemical character of AAs. Hydrophobic AA precursors like Cys, Phe, and Leu yielded CQDs of the highest QY. The lowest QY was found for hydrophilic Ser- and Asp-CQDs. Interestingly, a QY similar to that of Ser-CQDs and Asp-CQDs was determined for hydrophobic Pro-CQDs. The lower QY-value in this case can be connected to the smaller volume of the side chain. This is probably also the reason why the CQD derived from Leu containing a branched chain displayed a higher QY than the Gly-CQD.

**Table 1:** Fluorescence (λ_ex_ = 350 nm) of CQDs versus References

compound/AA-CQD	λ_em_, nm	QY, %^a^	QY, %^b^
**Cys**	**419**	90 **±** 5	86 **±** 7
**Lys**	**420**	67 **±** 3	63 **±** 1
His	410	50 ± 3	48 ± 5
Ser	430	32 ± 2	30 ± 4
Gly	418	55 ± 4	52 ± 2
Pro	418	14 ± 1	13 ± 1
**Phe**	**425**	90 **±** 4	85 **±** 6
Asp	413	20 ± 2	19 ± 1
**Leu**	**409**	87 **±** 5	83 **±** 4

a QS as a standard (QY = 54%, λ_em_ = 439 nm).

b Coumarin 1 as a standard (QY = 59%; λ_em_ = 445 nm).

For Cys-CQD, one can observe a higher QY due to the presence of sulfur as the doping heteroatom. The existence of sulfur could introduce defect sites, which alters the energy states and creates additional transition ways for electrons in the band structure of CQDs; or due to the similar electronegativity of carbon and sulfur, sulfur atoms could replace some of the carbon atoms in the core, resulting in high QYs.^62^ Those results agree with the literature data (Table 2). The hydrophobic character and larger volume of the side chains generally enhance the QY. The aromatic moiety hinders the interactions with polar solvents and, as a consequence, simplifies the electronic transition from HOMO to LUMO within. Similarly, hydrophilic side chains increase the interaction strength, with polar solvents reducing the extent of electronic transitions and hence QY.

**Table 2:** Comparison of CQDs Prepared from Various AAs^a^

AAs	conditions	particle size, nm	zeta potential, mV	excitation wavelength, nm	QY, %	emission wavelength, nm	ref
Arg, Lys, His, Cys, Met	Microwave method, 700 W, 1–4 min, + CA	6–17	–7.45 to 4.1	313	12.9–75	407–433(λ_ex_ = 330 nm)	(63)
				345	2.5–89.5		
Gly, Trp	Hydrothermal method, 200 °C, 8–12 h, + glucose	2–5.4	–19 to 21	n.d.	22.7–24.2	447 (λ_ex_ = 220–400 nm)	(64)
Phe, Tyr, Trp, His, Leu, Glu, Arg, Cys, Gly	Hydrothermal method, 180 °C, 12 h, + CA	1.5–7.5	–54.8 to 1.5	350–380	25.5–62.1	440–463(λ_ex_ = 350–380 nm)	(65)
Arg	Microwave method, 1000 W, 2 min, + CA	1–10	–10	n.d.	n.d.	425 (λ_ex_ = 310–350 nm)	(66)
Gly, Lys, Ser	Hydrothermal method, 180 °C, 6 h, + CA	2.3–14.5	n.d.	360	10.6–12.3	410–450(λ_ex_ = 340–380 nm)	(67)
His	Microwave method, 700 W, 2.7 min, + H_3_PO_4_	1–4	n.d.	360	44.9	440 (λ_ex_ = 360 nm)	(68)
Asn	Pyrolysis method	2.9	n.d.	n.d.	n.d.	441 (λ_ex_ = 348 nm)	(69)
Cys	Microwave method, 4 min, + CA	2–4	n.d.	355	81–85	435–460(λ_ex_ = 300–400 nm)	(70)
Gly	Hydrothermal method, 300 °C, 2 h	2.1–3.1	n.d.	365	30.6	410 −580(λ_ex_ = 365–465nm)	(37)
Cys, Lys, His, Ser, Gly, Pro, Phe, Asp, Leu	Hydrothermal method, 180 °C, 24 h, + CA	0.2–100	–18.5 to 7	350	14–90	409–439(λ_ex_ = 350 nm)	this work

a Arg—arginine, Lys—lysine, His—histidine, Cys—cysteine, Met—methionine, CA—citric acid, Gly—glycine, Trp—tryptophan, Asp—aspartic acid, Glu—glutamic acid, Tyr—tyrosine, Gln—glutamine, Phe—phenylalanine, Leu—leucine, Ser—serine, Asn—asparagine, and Pro—proline; n.d.—no data.

Table 2 shows that AAs were frequently used as the synthetic precursors of CQDs. Nevertheless, most of the studies focused on the applications of CQDs (antibacterial agents,^66^ sensors of toxic metal ions^67^ or rutin,^69^ and cellular imaging agents^70^) rather than on the structural differences between CQDs and their origins. The role of the functional group was studied by Hsu and Chang,^37^ while Gly was used as the only AA CQD synthetic precursor. Despite this, they found that AAs were promising candidates for the synthesis of water-soluble and photoluminescent CQDs. These results became an inspiration for other researchers. Similar trends were indicated by Jiang et al.^68^ Sahiner et al. prepared CQDs using a microwave assisted method. For the synthesis of CQDs, they used two types of AAs: those with positively charged side chains (Arg, Lys, and His) and those containing sulfur (Cys and Met). Cys-CQDs displayed the highest QY; however, no prospective results were achieved for Met-CQDs. This is probably a consequence of the insufficient incorporation of −SH groups into the CQD structure. For Cys-CQDs, the zeta potential was negative because of the presence of thiols of the lowest isoelectric point. For Met-CQDs, this value was positive, which may suggest a lower functionalization with −SH groups. This, in turn, can be connected with the lower S/C mass ratio and a higher thermal stability for Met.^63^ Yan et al. designed CQDs exhibiting three excitation peaks and excitation-independent emission. Apart from Trp and Gly, glucose was used as the precursor, and CQDs were tested toward the selective detection of Al^3+^.^64^ The most comprehensive studies of CQDs synthesized using AAs were performed by Pandit et al.,^65^ where CQDs were synthesized via a hydrothermal method in the presence of CA. Nonetheless, it should be emphasized that the QY for the so-obtained CQDs is far from the results presented in our work. The differences in QY could be the consequence of the proposed mechanism of the polymerization-carbonization process during hydrothermal synthesis. The growth of CQDs could be described by a competing generation of oligomers and carbonization. Hence, the composition of CQD depends on the reaction temperature and time because those parameters affect the number of polymeric structures, the appearance of microcrystalline regions or lattices, and the consumption of the polymer for core building. Zeng et al. showed that for the same substrates, one could obtain polymer chains/carbon structures or highly carbonized CQDs and spherical particles with an amorphous core or graphitic carbogenic particles.^71^ In summary, the differences in temperature and time influence the carbonization degree and, as a consequence, the optical CQD properties.

**Figure 6:**
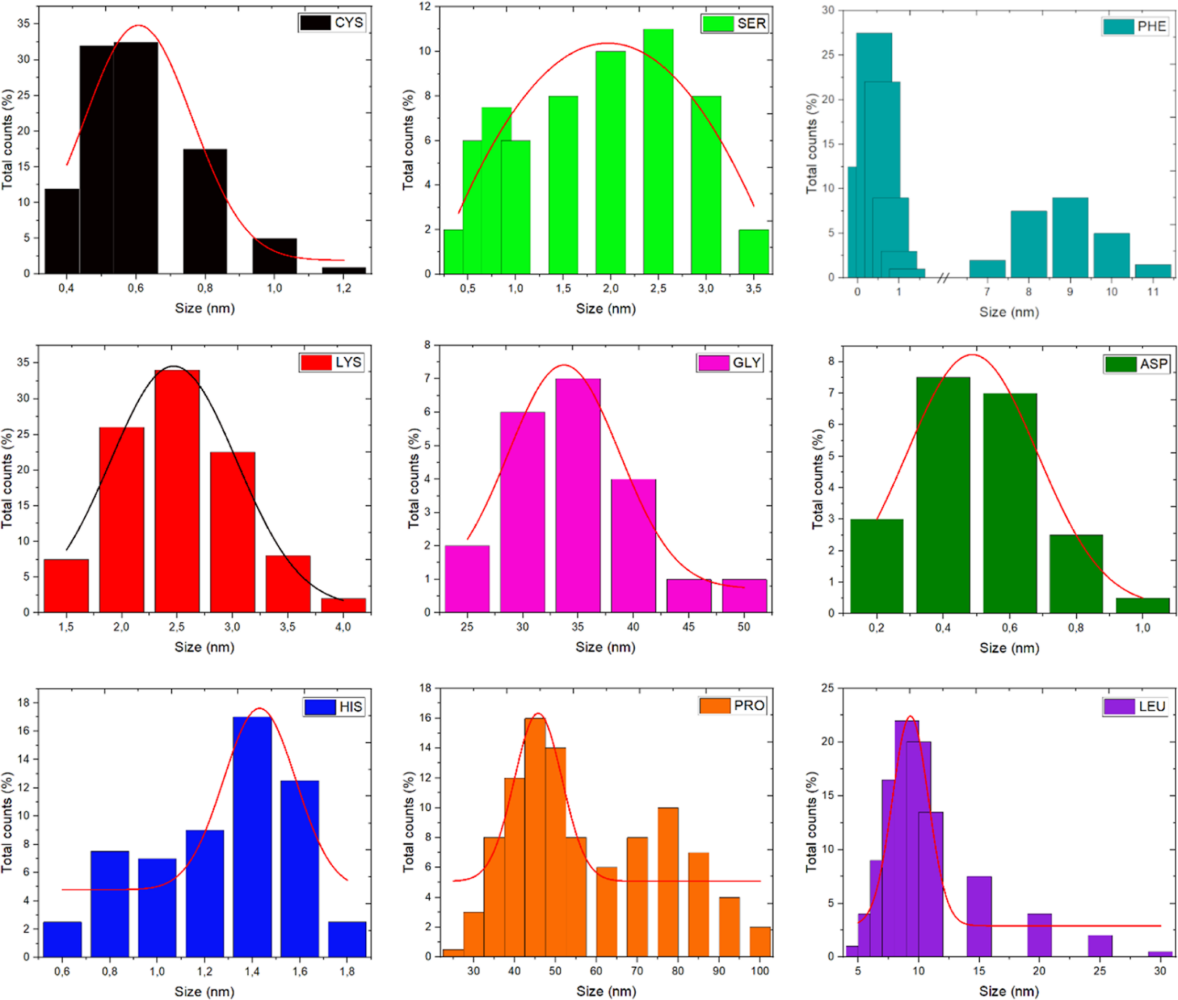
DLS data of the CQD volume–size distribution in the aqueous suspension under neutral pH; solid lines represent unimodal distribution curves.

Last but not least, for water-based applicabilities, DLS analysis was performed to determine the average size of CQDs and the stability of CQD aqueous dispersions (Figure 6). The diameters were found in the range of 0.2 to 100 nm. The dispersion containing the smallest CQDs, also with the lowest size distribution, was prepared from Cys-CQDs. Ser-, His-, and Asp-CQD dispersions also contained a small amount of larger particles. The lowest content of particles smaller than 10 nm was observed for Gly (only 21 vol %).

In colloids, the zeta potential is the difference between the potential of the outer mobile and the inner stationary layer attached to the particle dispersed in the continuous phase and can be considered as an indicator of dispersion stability. Samples with a high absolute value of zeta potential are electrically stabilized by repulsion, while those with low zeta potential tend to coagulate or flocculate. In the case of CQDs, multiple surface functional groups can improve the dispersion of CQDs in aqueous or, generally, polar solvents. And so, practically all CQDs had a negative zeta potential at neutral pH (Figure S5). This fact indicates that the CQD surfaces were rich in ionizable, negatively charged moieties like carboxylic (or thiol, etc.) groups, which fully corresponds to the previous analyses. The highest absolute values of the zeta potential in the broadest pH scale were found for Lys- and Cys-CQDs, providing excellent dispersibility and stability in water. At pH = 7, for His-, Leu-, Asp-, and Phe-CQDs, zeta potential values were almost neutral, while for Pro-CQDs, the zeta potential was positive. At pH = 2, most of the amine groups were protonated, giving the overall higher positive surface charge. At alkaline suspension, the zeta potential remains highly negative to reflect the presence of stable anions for all CQDs.

## Conclusions

CQDs obtained from sustainable sources such as AAs and via a green hydrothermal method represent an excellent class of application-tunable carbon nanomaterials. Here, the structural characterization and spectral properties of CQDs have been studied. The blue (and green) fluorescent CQDs were obtained without a purification step, while Cys-, Phe-, Leu-, and Lys-CQDs showed high QYs, conquering the conventional dyes. It was found that the structure of AAs had a great impact on the optical properties of CQDs, such as emission wavelength, excitation wavelength-dependent fluorescence, and QY. Moreover, the water stability of Lys-CQDs (and to a lesser extent, Cys-CQDs) was not compromised by extreme pH environments.

Despite the promising results listed above, it must be emphasized that future research must address, if synthesized in a versatile and economic approach from sustainable sources, the separation of CQDs by size. The separation step and covalent functionalization with a well-defined linker via, for example, carboxylic groups, should lead not only to a narrower size distribution of water-soluble and water-stable CQDs but also, first of all, allow for full-color fluorescence without changing the excitation wavelength as the most pressing requirement toward programmable fluorescent probes and catalysts—only to mention the most ready-to-scaleup applications.

## Supporting Information Available

Representative TEM and SAED images of CQDs, fluorescence spectra of CQDs as a function of the exciting wavelength from 200 to 480 nm, and zeta potential of CQD dispersions at various pH values for zeta potential distribution(PDF)
