# Supplementary material for: Carbon Quantum Dots from Amino Acids Revisited: Survey of Renewable Precursors toward High Quantum-Yield Blue and Green Fluorescence
Source: ACS Omega. 2022 Nov 1;7(45):41165–76. doi: 10.1021/acsomega.2c04751 (PMC9670729; doi:10.1021/acsomega.2c04751)
Supplement: Supplementary file 1 — ao2c04751_si_001.pdf [file ao2c04751_si_001.pdf]

Supporting Information

for

Carbon Quantum Dots from Amino Acids Revisited:

Survey of Renewable Precursors

toward High Quantum Yield Blue and Green Fluorescence

Anna Kolanowska[1,2,3]*, Grzegorz Dzido[4], Maciej Krzywiecki[5], Mateusz M. Tomczyk[2], Dariusz Łukowiec[6], Szymon Ruczka[1,7], Sławomir Boncel[1,7,]*

[1] Silesian University of Technology, Faculty of Chemistry, Department of Organic Chemistry, Bioorganic Chemistry and Biotechnology, 44-100 Gliwice, Poland

[2] Silesian University of Technology, Faculty of Chemistry, Department of Physical Chemistry and Technology of Polymers, 44-100 Gliwice, Poland

[3] Biotechnology Centre, Silesian University of Technology, Krzywoustego 8, 44-100 Gliwice, Poland

[4] Silesian University of Technology, Faculty of Chemistry, Department of Chemical Engineering and Process Design, 44-100 Gliwice, Poland

[5] Silesian University of Technology, Institute of Physics—CSE, Konarskiego 22B, 44-100 Gliwice, Poland

[6] Silesian University of Technology, Materials Research Laboratory, Faculty of Mechanical Engineering, Konarskiego 18A, 44-100 Gliwice, Poland

[7] Silesian University of Technology, Centre for Organic and Nanohybrid Electronics, Konarskiego 22B, 44-100 Gliwice, Poland

*Corresponding authors: Anna Kolanowska, anna.kolanowska@polsl.pl; Sławomir Boncel slawomir.boncel@polsl.pl.

Supporting Information is presented on five (5) pages (S1-S5), and contains 5 figures (**Figure S1-S5**).

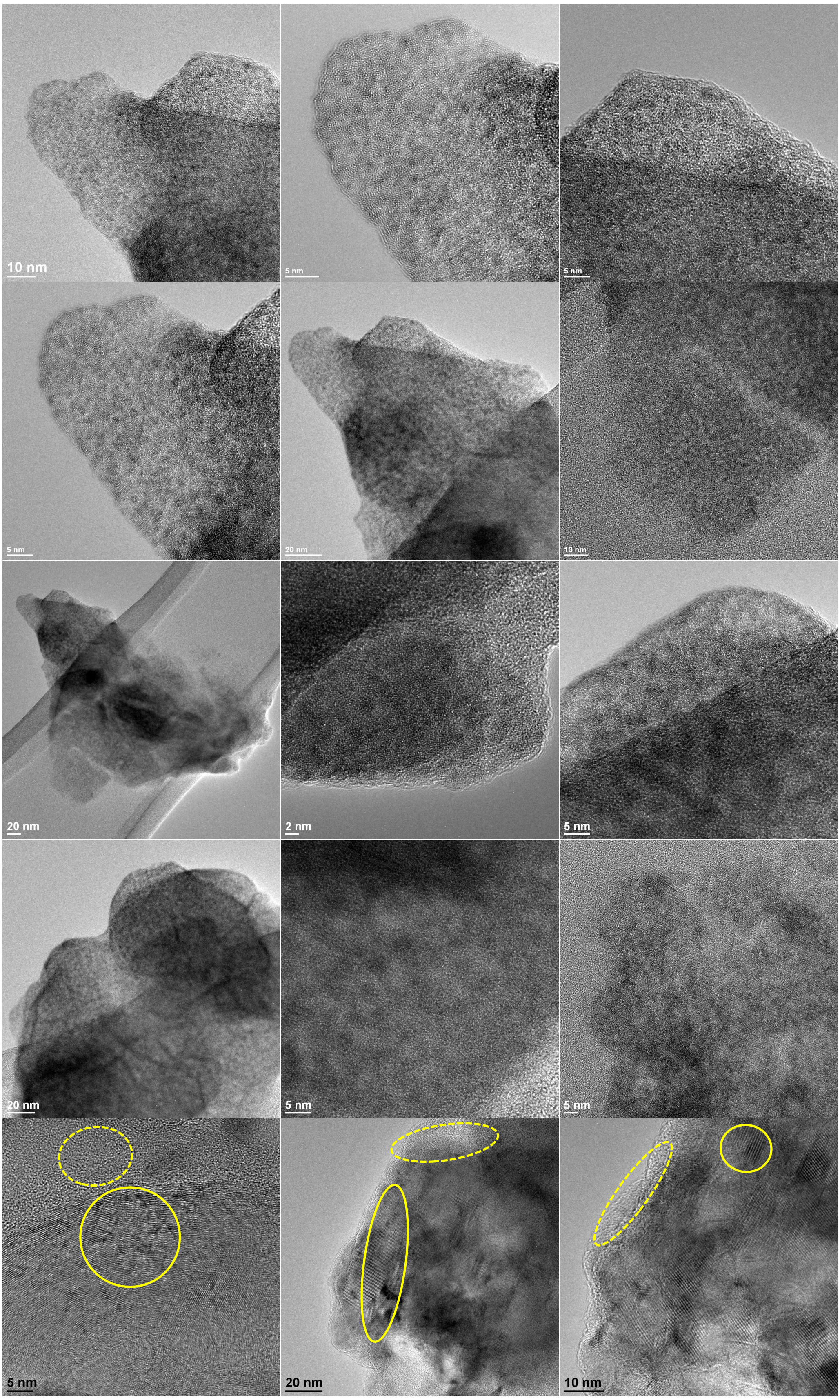

Figure S1 TEM images of the representative CQDs revealing their size and morphology; highlighted amorphous (dashed) and semi-graphitic polycrystalline (solid) domains

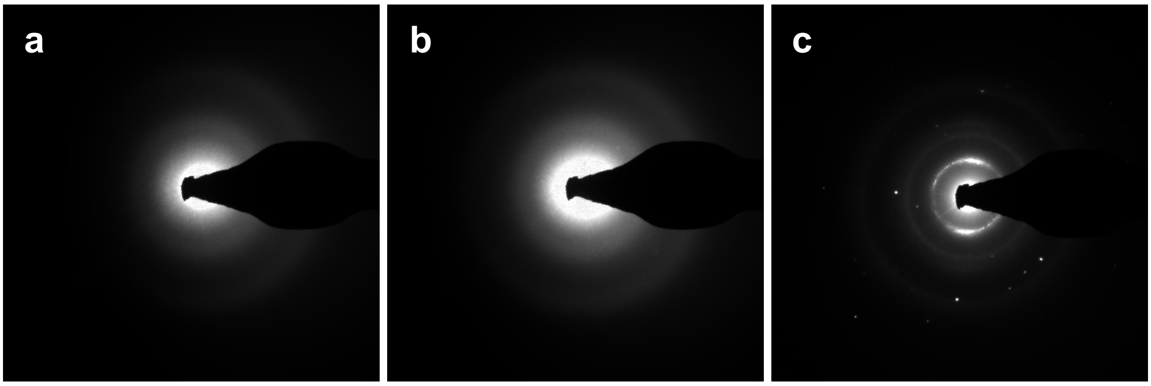

Figure S2 SAED patterns of the representative CQDs revealing amorphous (a, b) and semi-graphitic, polycrystalline domains (c)

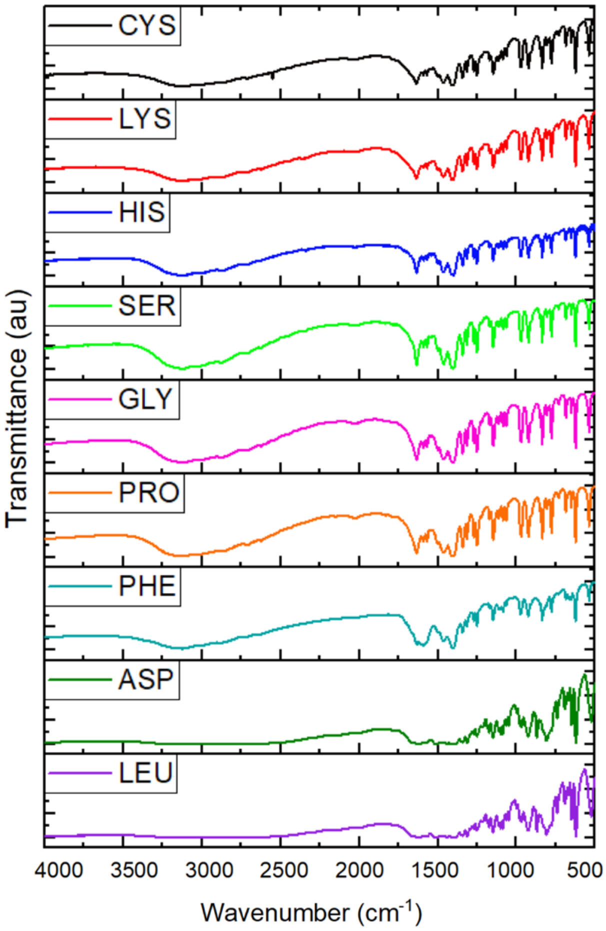

Figure S3 FT-IR spectra of all CQDs

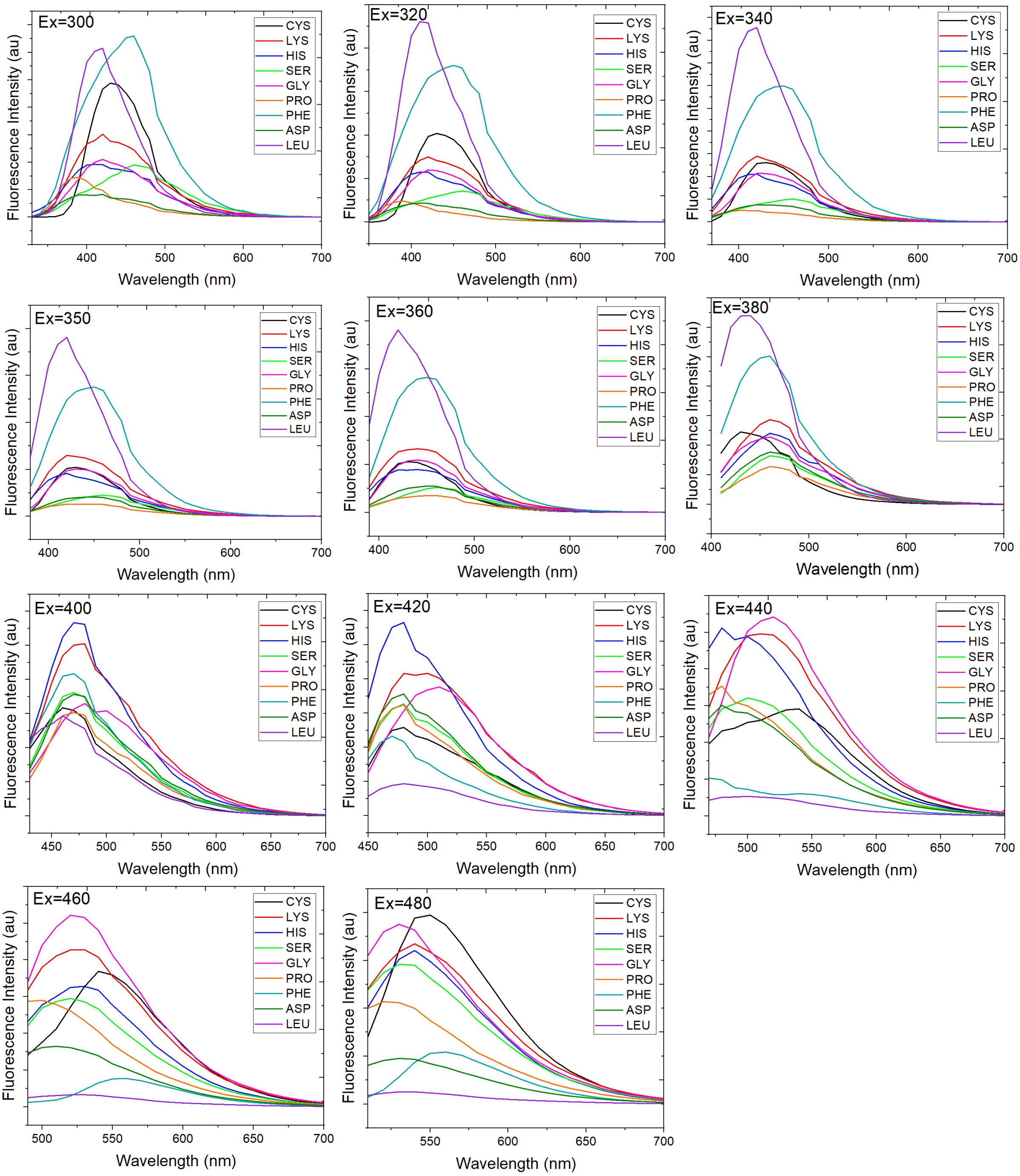

Figure S4 Fluorescence spectra of CQDs as a function of the exciting wavelength from 200 to 480 nm.

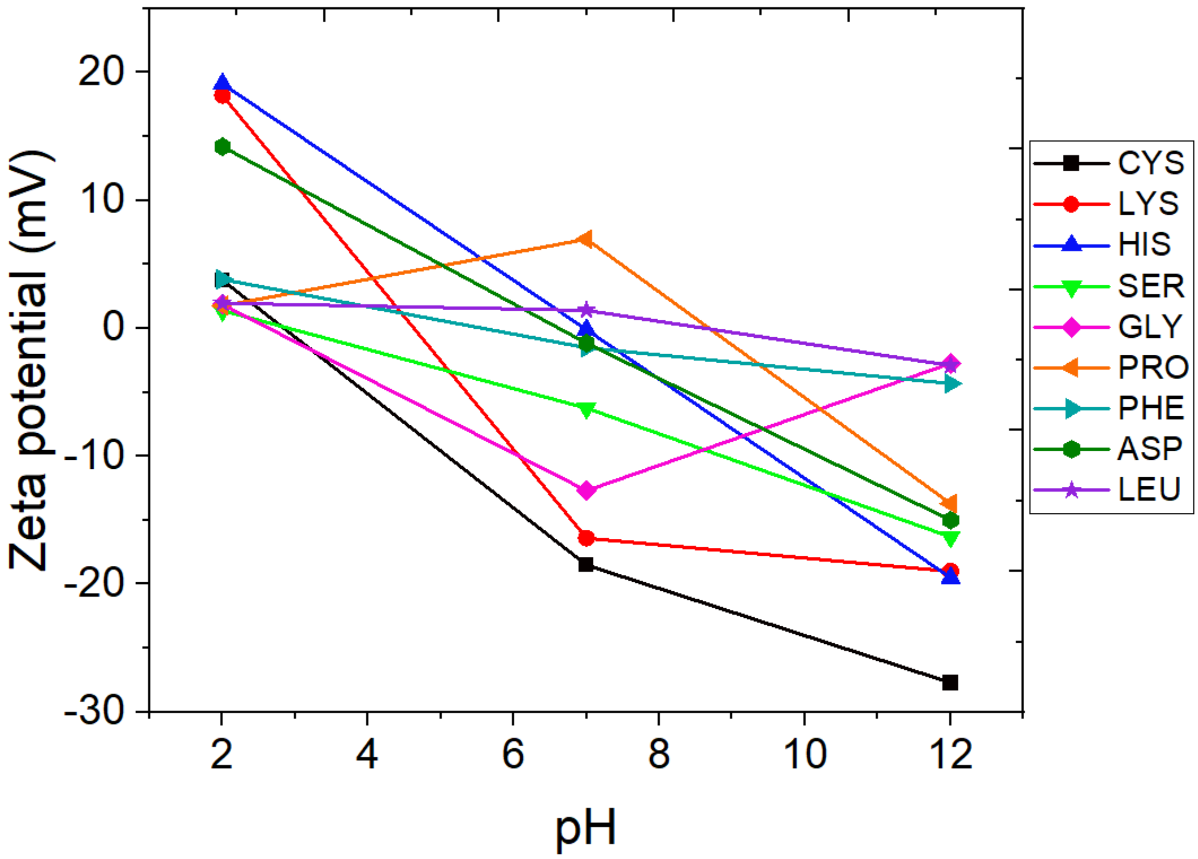

pH	Zeta potential [mV]								
	Cys	Lys	His	Ser	Gly	Pro	Phe	Asp	Leu
2	3.8 ±0.7	18.2 ±0.6	19.1 ±2.1	1.4 ±0.6	1.9 ±0.2	1.8 ±0.6	3.8 ±0.8	14.2 ±2.4	2.0 ±0.9
7	-18.5 ±1.1	-16.4 ±0.2	-0.1 ±0.01	-6.2 ±0.3	-12.6 ±1.8	7.0 ±0.7	-1.5 ±0.9	-1.2 ±0.9	1.4 ±0.4
12	-27.7 ±0.6	-19.0 ±1.4	-19.5 ±3.4	-16.3 ±2.8	-2.7 ±0.3	-13.7 ±4.4	-4.3 ±0.8	-15 ±4.1	-2.9 ±0.4

Figure S5 Zeta potential of CQD dispersions at various pH values; the solid lines represent only eye-guidelines
